# Advanced Engineering of Lipid Metabolism in *Nicotiana benthamiana* Using a Draft Genome and the V2 Viral Silencing-Suppressor Protein

**DOI:** 10.1371/journal.pone.0052717

**Published:** 2012-12-26

**Authors:** Fatima Naim, Kenlee Nakasugi, Ross N. Crowhurst, Elena Hilario, Alexander B. Zwart, Roger P. Hellens, Jennifer M. Taylor, Peter M. Waterhouse, Craig C. Wood

**Affiliations:** 1 Plant Industry, The Commonwealth Scientific and Industrial Research Organisation, Canberra, Australian Capital Territory, Australia; 2 School of Biological Sciences, University of Sydney, Sydney, New South Wales, Australia; 3 Breeding and Genomics, The New Zealand Institute for Plant and Food Research Limited, Mt Albert, Auckland, New Zealand; 4 Mathematics, Informatics and Statistics, The Commonwealth Scientific and Industrial Research Organisation, Canberra, Australian Capital Territory, Australia; 5 School of Molecular Bioscience, School of Biological Sciences, University of Sydney, Sydney, New South Wales, Australia; Centro de Investigación y de Estudios Avanzados del IPN, Mexico

## Abstract

The transient leaf assay in *Nicotiana benthamiana* is widely used in plant sciences, with one application being the rapid assembly of complex multigene pathways that produce new fatty acid profiles. This rapid and facile assay would be further improved if it were possible to simultaneously overexpress transgenes while accurately silencing endogenes. Here, we report a draft genome resource for *N. benthamiana* spanning over 75% of the 3.1 Gb haploid genome. This resource revealed a two-member *NbFAD2* family, *NbFAD2.1* and *NbFAD2.2*, and quantitative RT-PCR (qRT-PCR) confirmed their expression in leaves. FAD2 activities were silenced using hairpin RNAi as monitored by qRT-PCR and biochemical assays. Silencing of endogenous FAD2 activities was combined with overexpression of transgenes via the use of the alternative viral silencing-suppressor protein, V2, from *Tomato yellow leaf curl virus*. We show that V2 permits maximal overexpression of transgenes but, crucially, also allows hairpin RNAi to operate unimpeded. To illustrate the efficacy of the V2-based leaf assay system, endogenous lipids were shunted from the desaturation of 18∶1 to elongation reactions beginning with 18∶1 as substrate. These V2-based leaf assays produced ∼50% more elongated fatty acid products than p19-based assays. Analyses of small RNA populations generated from hairpin RNAi against *NbFAD2* confirm that the siRNA population is dominated by 21 and 22 nt species derived from the hairpin. Collectively, these new tools expand the range of uses and possibilities for metabolic engineering in transient leaf assays.

## Introduction

*Nicotiana benthamiana* is widely used in the plant sciences in both basic and applied contexts. It has long been used as a host in which to study plant-pathogen interactions [1], however with the advent of enhanced transient leaf assays [2] it has been adopted for metabolic engineering [3]. The enhancement of transgene expression in agro-infiltration leaf assays as described by Voinnet and colleagues uses the viral silencing-suppressor protein (VSP), p19, from the *Tomato bushy stunt virus* to interfere with the host’s endogenous transgene silencing pathway, thus allowing co-infiltrated transgenes to express at high levels for extended periods. This enhanced leaf assay format permits complex multigene pathways to be assembled ‘in planta’ from individual T-DNA expression vectors allowing their pathway activities to be assessed within a few days [3], [4], [5], [6]. Such an assay format allows individual components of complex pathways to be readily interchanged and compared side-by-side on a single leaf. Such comparisons can guide the design and composition of large single vector constructs that may then be deployed for stable expression in plants [7].

Plant viruses have evolved a diverse array of strategies to evade the host’s viral-defence apparatus, including a range of VSPs that target different components of the RNAi machinery [8]. p19 is perhaps the best studied VSP with crystal structures showing that it forms a homo-dimer that binds to the duplexed form of small interfering RNAs (siRNAs) [9], [10]. V2, a VSP isolated from *Tomato yellow leaf curl virus* [11], [12], [13], either directly binds to the plant-encoded SGS3 protein [14] or the double-stranded RNA structures with 5′ overhangs which are involved in the generation of siRNAs from single stranded RNA by the co-suppression pathway [12], [15], [16]. Here we sought to investigate the use of V2 to inhibit the production of siRNA from the single-stranded mRNA of agro-infiltrated transgenes, while allowing siRNAs derived from infiltrated hairpin (hp) RNA constructs to operate unhindered.

Although many transgenic pathways have been overexpressed in transient assays, the background host metabolism is often less than ideal for maximal flux into the desired endpoints. The lipid species oleic acid, 18∶1, is an example of a metabolite that is rapidly converted into linoleic acid, 18∶2, via endogenous desaturase activity, FAD2, thus preventing 18∶1 from entering a range of other engineered pathways [17]. Therefore, the silencing of FAD2 activity would allow a build up of 18∶1 in leaf assays suitable for further complex modifications. In the present study we use the fatty acid elongase from *Arabidopsis thaliana* [18], *AtFAE1*, to catalyse the conversion of 18∶1 to gondoic acid (20∶1) in leaf assays. Gondoic acid is barely detectable in *N. benthamiana* leaves, and therefore the accumulation of 20∶1 allows monitoring of the flux of endogenous lipid species into new pathways. Similarly *N. benthamiana* leaves produce little oil, however the overexpression of *AtDGAT1*, a diacylglycerol-O-acyl transferase from *A. thaliana*, shunts endogenous lipids into oil [3] that are readily quantified. The study of modified oils produced in non-seed tissues, such as leaves, may provide more renewable biofuels and help in the effort to relieve the world’s current reliance on fossil fuels [19].

Despite being a commonly-used model plant [1] there are scant genomic resources for *N. benthamiana*. Silencing of endogenes, such as FAD2, in *N. benthamiana* would be greatly facilitated by improved genomic resources of this model plant. *N. benthamiana* has a ∼3.1 Gb haploid genome that, if fully resolved, would provide a useful resource for accurate genetic engineering of trangenic pathways. Here we use next generation sequencing technologies to assemble a draft genome sequence of *N. benthamiana* that assisted in gene discovery and the accurate design of silencing constructs. Furthermore, we outline a V2-based transient assay format that facilitates both high level transgene overexpression and simultaneous endogene silencing. The genome resource and the V2-based assays were used to design and engineer the production of high levels of leaf oils containing new fatty acids.

## Results

### V2 and p19 Enhance Transgene Overexpression in N. benthamiana Transient Assays to a Similar Degree

**Figure 1:**
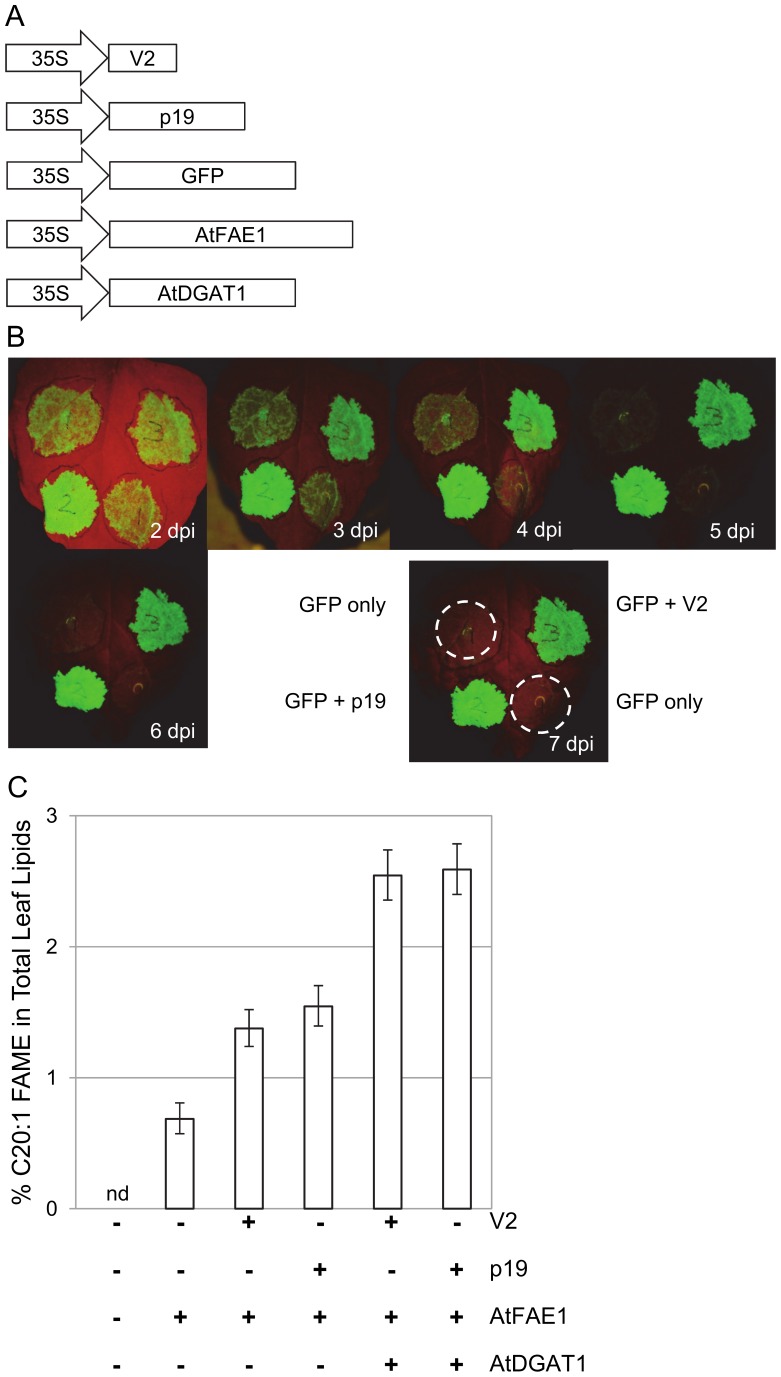
V2 supports the overexpression of multistep transgenic pathways in transient leaf assays. A. Schematic of T-DNA binary constructs for leaf expression of the viral silencing-suppressor proteins (VSP), V2 and p19, and the transgenes GFP, AtFAE1 and AtDGAT1. All ORF constructs were driven by the *Cauliflower mosaic virus* 35S promoter. **B.** Time course of GFP expression in transient leaf assays with either no VSP or co-infiltration of V2 or p19. Images show one representative leaf photographed daily from 2 to 7 days post infiltration (dpi), and the last image is used to indicate the placement of infiltrations with a stippled circle showing where complete co-suppression of GFP has occurred 7 dpi. **C.** Different combinations of VSPs and transgenes, *AtFAE1* and *AtDGAT1*, showing the increase in level of the elongated fatty acid 20∶1 in total leaf lipids. Data shown are the means and 5% LSD bars calculated from 10 samples. When the LSD bars for two metabolites fail to overlap, the treatments are significantly different at the 5% level of significance.

In transient leaf assays, p19 is often used to enhance transgene overexpression. We used this p19-mediated enhancement of transgene activity as the benchmark to compare against assays using V2. The coding regions of the VSPs were inserted into 35S-regulated binary expression plasmids (Fig. 1A) and agro-infiltrated in various combinations with green fluorescent protein (GFP) or fatty acid metabolic enzyme constructs into *N. benthamiana* leaves. Visual observations of the GFP-infiltrated leaves 7 days post infiltration (dpi) showed that V2 enhanced GFP expression with an efficacy similar to that of p19 (Fig. 1B). The suppressor activities were further quantified using overexpression constructs encoding fatty acid elongase 1 (AtFAE1) and acyl-CoA:diacylglycerol acyltransferase 1 (AtDGAT1) from *A. thaliana* (Fig. 1C). These enzymes catalyse the production of modified fatty acids, including the fatty acid, 20∶1 (gondoic acid) and production of triacyglycerides (TAG). When co-infiltrated with the *AtFAE1* construct, V2 and p19 increased the production of gondoic acid by 1.9 and 2 fold, respectively. With the further addition of *AtDGAT1*, the VSPs each raised the level of 20∶1 by approximately 3.5 fold (Fig. 1C). These results demonstrate that, although targeting different components of the plant’s silencing machinery, both VSPs give similar levels of protection against the co-suppression of transgene activities in *N. benthamiana* transient assays.

### V2 Permits hpRNA-mediated Silencing of Transiently-expressed GFP

**Figure 2:**
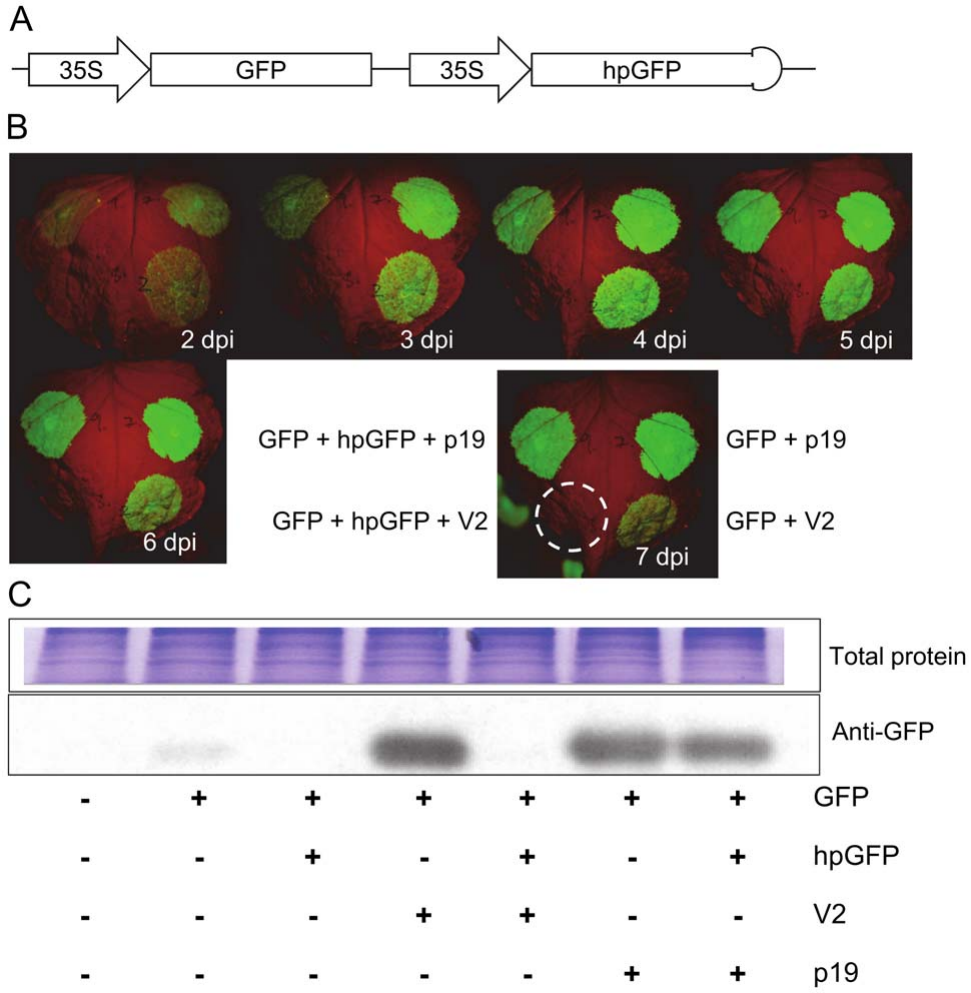
V2, not p19, allows efficient hairpin-based silencing of a co-infiltrated transgene, GFP. A. Schematic of the T-DNA binary construct containing a GFP expression cassette and a 380 bp hairpin directed against GFP, hpGFP. **B.** Time course of GFP expression in transient leaf assays infiltrated with combinations of GFP and hpGFP with V2 or p19. Images show one representative leaf photographed daily from 2 to 7 days post infiltration (dpi), and the last image is used to indicate the placement of infiltrations with stippled circles showing infiltration zones that failed to express GFP. **C.** Western blot analysis of GFP expression in *N. benthamiana* leaves infiltrated with combinations of GFP, hpGFP, V2 and p19 as indicated. Samples were collected 4 dpi. The upper panel shows the total protein loaded for the western blot and the lower panel shows the signal generated from an antibody recognising GFP, anti-GFP.

V2 suppresses the plant’s co-suppression silencing pathway by interacting either directly or indirectly with SGS3 [11], [12] but hpRNA-mediated silencing [20] operates effectively in *sgs3* mutant plants [15]. Therefore, it seemed possible that while the expression of V2 in transient assays could be used to enhance the expression of a delivered transgene, it may, unlike p19, permit hpRNA-mediated silencing of targeted genes. To test this, the efficacy of a hpRNA targeting GFP was evaluated in transient leaf assays in the presence of either V2 or p19 (Fig. 2). In concordance with previous reports [2], [21], [22], [23], the silencing of GFP by a hpRNA construct was prevented by co-expression of p19 (Fig. 2B). In contrast, the hpRNA-mediated silencing remained effective in the presence of V2 (Fig. 2B). This dichotomy of suppressive behaviour was confirmed by immuno-blots measuring GFP protein levels (Fig. 2C) and supported the notion that in transient leaf assays V2 could facilitate strong overexpression of transgenes yet allow concurrent hpRNA-directed silencing.

### A Draft Genome Assembly for N. benthamiana

Metabolic engineering in *N. benthamiana* would be improved with better knowledge of the genome, allowing more accurate design of hairpin RNAi silencing molecules and the discovery and characterisation of gene families. We used next-generation sequencing of *N. benthamiana* nuclear genomic DNA to generate 836,954,626 paired-end short-read sequences on an Illumina HiSeq2000™ instrument representing ∼48× coverage of the estimated 3.1 Gb genome size (Table 1). The draft genome assembly yielded 275,036 scaffolds covering 76.4% of the estimated haploid genome with a longest scaffold of 447 kb, N50 of 31.8 kb, and N90 of 6.32 kb (Table 2). A BLAST-searchable database of this assembly is available at www.benthgenome.com.

**Table 1:** Summary of sequencing libraries generated from nuclear genomic DNA of *N. benthamiana.*

PE Libraries	Estimated Insert Size	Reads Pairs	Trimming Undertaken	Total Bases Available After Trimming	Estimate Genome Coverage (%)
PE180-L1	194	94,803,174	not required	18,960,634,800	5.93
PE180-L2	194	81,459,743	not required	16,291,948,600	5.09
PE300-L4	363	125,192,660	10 bases 5 prime end removed	22,534,678,800	7.04
PE500-L5	363	106,570,812	10 bases 5 prime end removed	19,182,746,160	5.99
PE500-L2	444	124,041,289	10 bases 5 prime end removed	22,327,432,020	6.98
PE500-L3	444	81,765,307	10 bases 5 prime end removed	14,717,755,260	4.60
PE500-L6	450	111,876,143	10 bases 5 prime end removed	20,137,705,740	6.29
PE500-L7	450	111,245,498	10 bases 5 prime end removed	20,024,189,640	6.26
Total PE Reads		836,954,626		154,177,091,020	48.18
**LIMP Libraries**	**Insert Size**	**Reads Pairs**	**Trimming**		
LIMP2k-L1	2000	51,387,190	Trimmed to 36 bases and duplicates removed		

**Table 2:** Summary of draft assembly of *N. benthamiana.*

	Contigs	Scaffolds
Number	300,384	275,036
Total size (bases)	2,440,560,8830	2,443,539,138
Total as % of estimated genome size	76.3	76.4
Percentage of Ns present	0.00	0.13
Longest	307,106	447,128
Shortest	8	201
Mean size	8,125	8,884
Median size	1,071	1,385
N90 length	6,179	6,327
N75 length	15,235	15,538
N50 length	31,255	31,834
N25 length	55,660	56,670
L50 count	22,438	22,068
NG50 length	20,869	21,350
LG50 count	37,284	36,552
Percentage of assembly in scaffolded contigs		7.6
Percentage of assembly in unscaffolded contigs		92.4
Average number of contigs per scaffold		1.1

As silencing of endogenes is an important aspect of metabolic engineering, we searched this genome resource for *N. benthamiana* sequences homologous to *A. thaliana FAD2*. This search revealed two *NbFAD2* genes, here named *NbFAD2.1* and *NbFAD2.2*. Both genes were subsequently cloned via PCR amplification from a DNA template and verified via conventional long-read sequencing (data not shown). Both *NbFAD2* genes contain the canonical histidine box motifs essential for 18∶1 desaturation activity [16]. Real-time PCR analysis demonstrated that *NbFAD2.2* was expressed at ∼40% higher level than *NbFAD2.1* in leaves used in infiltration experiments (Fig. S1), however *NbFAD2.1* was strongly expressed in developing seed (data not shown). *NbFAD2.1* and *NbFAD2.2* are 73% identical across the entire sequence and share regions of ∼50 bp length with greater than 90% similarity at the DNA level across the histidine box motifs.

### Silencing NbFAD2 Activity via Hairpin RNAi

**Figure 3:**
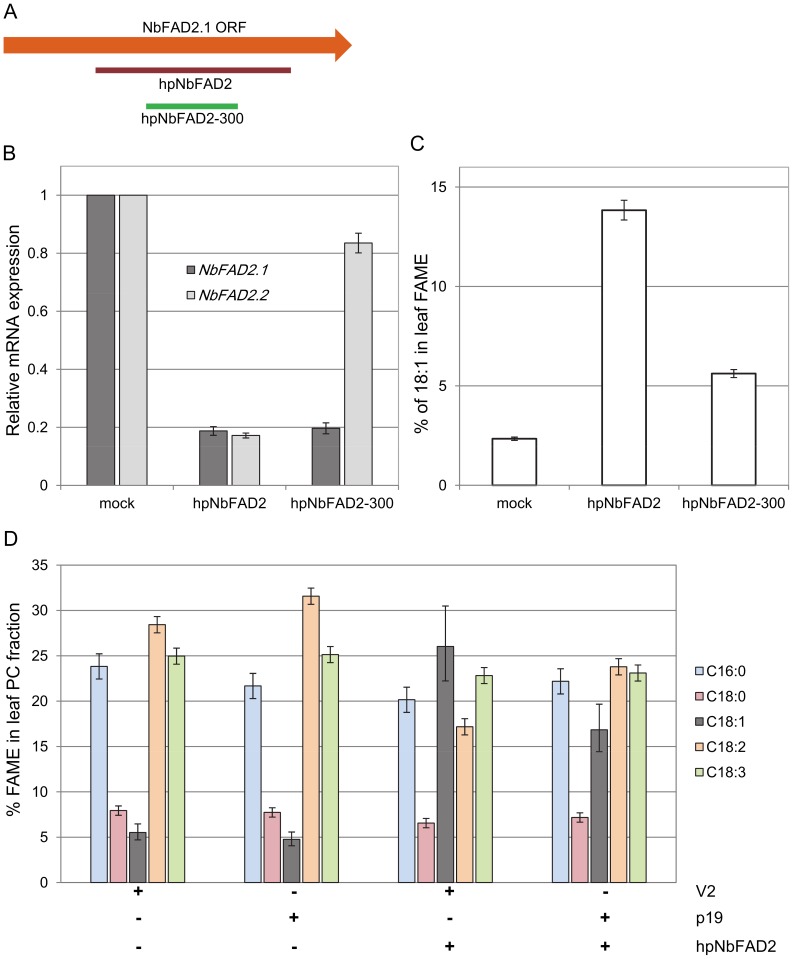
Effective silencing of the FAD2 gene family in *N. benthamiana* leaves. A. Relative position and length of the 660 bp hairpin, hpNbFAD2, and the 300 bp hairpin, hpNbFAD2-300, against *NbFAD2.1*. Higher resolution alignments are found in Figure S2. **B.** Reduction in expression levels of *NbFAD2.1* and *NbFAD2.2* when silenced using hpNbFAD2 and hpNbFAD2-300 hairpin RNA construct. Total RNA extracted from leaves infiltrated with the different hairpin constructs 4 days post infiltration and expression levels of *NbFAD2.1* and *NbFAD2.2* were analysed by quantitative real-time, qRT-PCR. Mock represents RNA extracted from leaf tissue infiltrated with agrobacterium only. Data shown are means of 3 biological replicates with 3 technical repeats for each and error bars are the standard errors of the mean. **C.** Total fatty acid analysis of leaves infiltrated with either the hpNbFAD2 and hpNbFAD2-300 hairpin. Fatty acid methyl esters (FAME) were analysed 4 days post infiltration and the data shown are the mean and 5% LSD bars calculated from 10 independent leaf infiltrations per treatment. When the LSD bars for two metabolites fail to overlap, the metabolite means may be regarded as significantly different at the 5% level of significance. Mock refers to leaves infiltrated with *Agrobacterium* only. **D.** Fatty acid profile of phosphatidylcholine (PC) fraction extracted from leaves infiltrated with combinations of V2, p19 and hpNbFAD2. Data shown are the mean and 5% LSD bars calculated from at least 5 infiltrations. When the LSD bars for two metabolites fail to overlap, the treatments may be regarded as significantly different at the 5% level of significance.

To reduce both NbFAD2 activities in transient assays a 660 bp hpRNA, hpNbFAD2, was designed that spanned the highly conserved regions of both *NbFAD2.1* and *NbFAD2.2*. A shorter hairpin, hpNbFAD2-300, was designed against a central portion of *NbFAD2.1* which is more divergent in sequence from *NbFAD2.2* (Fig. 3A). Both hpNbFAD2 and hpNbFAD2-300 were infiltrated in leaves and quantitative real-time PCR (qRT-PCR) analyses demonstrated that both *NbFAD2.1* and *NbFAD2.2* were silenced by infiltration of hpNbFAD2 (Fig. 3B). The shorter hairpin, hpNbFAD2-300, silenced *NbFAD2.1* gene expression to the same degree as the longer hairpin, but was much more specific against *NbFAD2.1*, being only partially active in silencing *NbFAD2.2* gene expression (Fig. 3B). The leaves infiltrated with hpNbFAD2 and hpNbFAD2-300 were also assessed for changes in their fatty acid profiles. Total leaf lipids were extracted from infiltrated leaves and the changes in the level of 18∶1 suggest that hpNbFAD2 is more effective in increasing 18∶1 from 2.3% in mock control leaves to 13.8% in leaves infiltrated with hpNbFAD2. hpNbFAD2-300 partially silences NbFAD2 activities, increasing 18∶1 to 5.6%, a result consistent with the qRT-PCR results.

FAD2 desaturases are active on the phosphatidylcholine (PC) lipid species and the fatty acid composition of the PC fraction was analysed in leaves infiltrated with combinations of hpNbFAD2 and either V2 or p19 (Fig. 3D). The combination of hpNbFAD2 and V2 increases the 18∶1 content on PC to over 25%, whereas the combination of p19 and hpNbFAD2 increased 18∶1 content to ∼17%. The increase in 18∶1 content is mostly reflected in a decrease in the 18∶2 content on PC, and the other major fatty acids were largely unaffected.

### Deep Sequencing Analysis of the Small RNA Population Generated by hpNbFAD2

**Figure 4:**
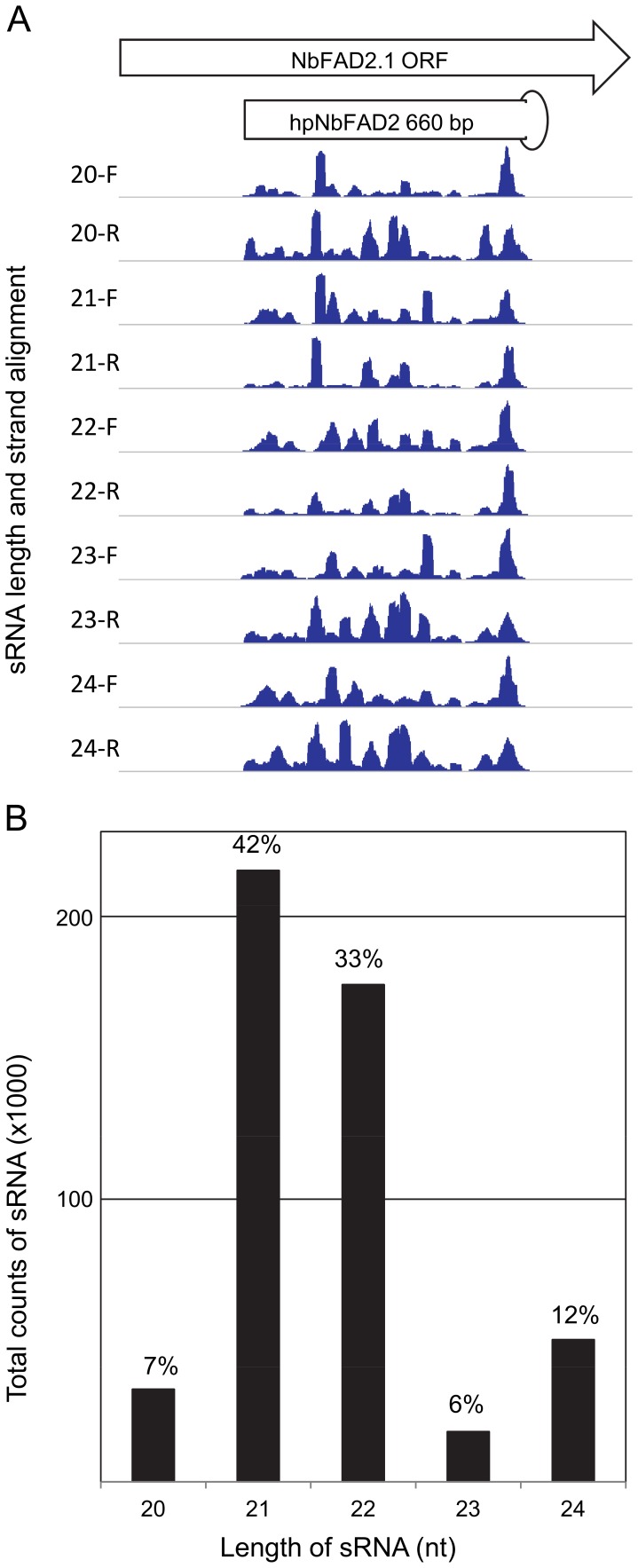
Size and distribution of sRNA populations generated by a hairpin targeting the endogene *NbFAD2.1*. **A.** The ORF of *NbFAD2.1* is portrayed indicating the region used to generate a 660 bp hairpin, hpNbFAD2. Total RNA was extracted from leaves infiltrated with hpNbFAD2 4 days post infiltration. The size and distribution of the dominate classes of small RNAs on the forward (F) and reverse (R) strand of the *NbFAD2.1* is illustrated. Absolute read coverage is normalised for each track. **B.** Absolute numbers and relative percentages of the dominant sRNA size classes generated by hpNbFAD2.

The sRNAs generated by infiltration of leaves with the 660 bp hpNbFAD2 were analysed by ‘deep’ sequencing approaches (Fig. 4). This showed that small RNA (sRNA) populations generated from the transient overexpression of the 660 bp hairpin RNA, were exclusively confined to the region of the hairpin, with no sRNA reads mapping to *NbFAD2.1* outside of the primary target region (Fig. 4A). The sRNA populations mapped unevenly across the target region of the hairpin and the distribution and abundance of the reads were different for each size class (20–24 nt). The 21 and 22 nt size classes dominated the overall profile in either orientation (Fig. 4B) and accounted for approximately 42% and 33%, respectively, of the population analysed.

### Shunting Endogenous Lipid Metabolites into Engineered Transgenic Pathways Using a V2-mediated Assay Format

**Figure 5:**
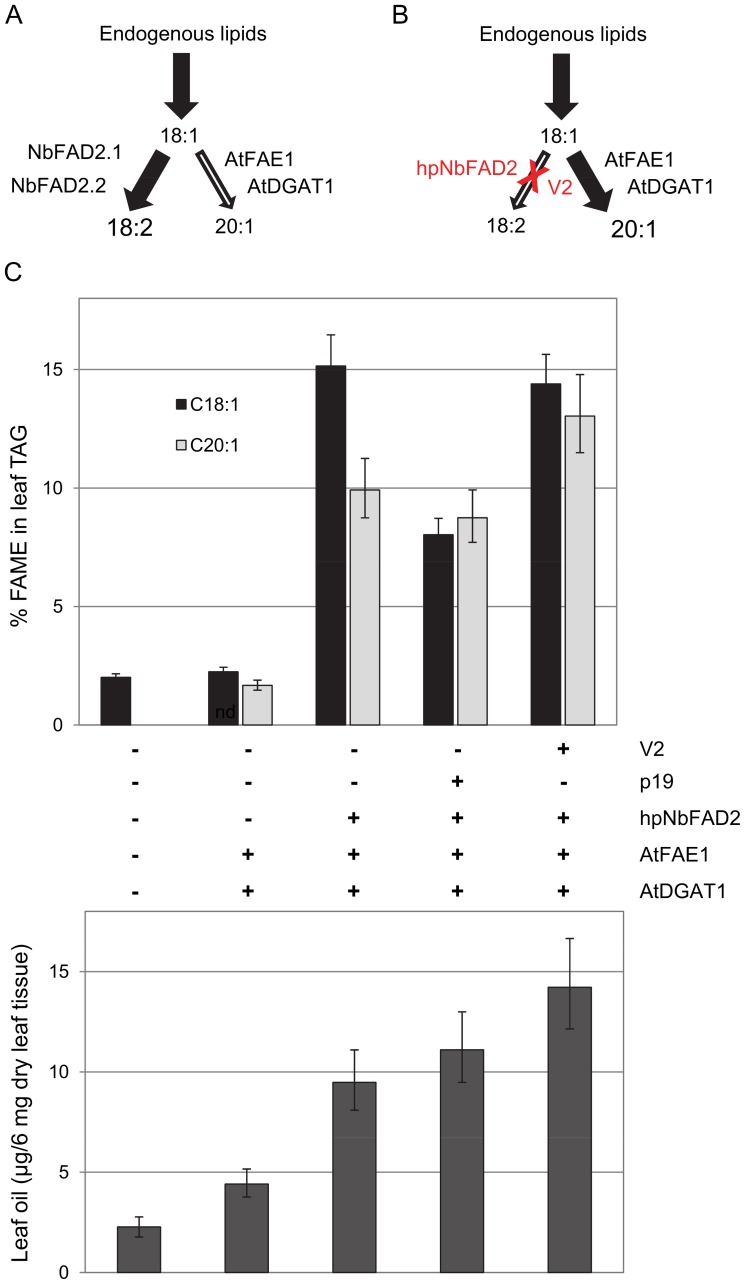
Silencing endogene in transient leaf assays to improve the flux of metabolites into transgenic pathways. **A.** Diagram outlining the flux of lipid metabolites from 18∶1 to 18∶2, via NbFAD2 and limiting the availability of 18∶1 to enter into transgenic pathways such as that catalysed by AtFAE1 and AtDGAT1 to produce 20∶1. **B.** Diagram outlining the efficient RNAi (hpNbFAD2) silencing of endogenous NbFAD2 activities, to increase the flux of 18∶1 into the engineered pathway to produce 20∶1, whereby both silencing and transgene overexpression are supported with the addition of V2. **C.** Combinations of hpNbFAD2, V2 or p19, and a two step metabolic pathway, *AtFAE1* and *AtDGAT1*, for the production of oil (lower panel), and the weight percentage of 18∶1 and 20∶1 in the total FAME in the oil (upper panel). Data shown are the mean and 5% LSD bars calculated from at least 4–6 infiltrations. When the LSD bars for two metabolites fail to overlap, treatments are significantly different at the 5% level of significance. The complete fatty acid profiles of leaf oils from this experiment are found in Table S1.

An example of metabolic engineering is outlined in Fig. 5A & 5B, where the steady state flux of lipids in the host plant is shunted into a transgene-engineered pathway by the concomitant silencing of an endogene. The hpNbFAD2 construct was used to reduce all NbFAD2 activities in infiltrated *N. benthamiana* leaves and elevate 18∶1 levels. Combinations of the hpNbFAD2 and overexpression constructs AtFAE1, AtDGAT1, p19 and or V2, were agro-infiltrated into *N. benthamiana* leaves and the production of leaf oils and their composition analysed (Fig. 5C). The composition of the leaf oils is a reflection of the AtFAE1 activity and the amount of leaf oil is dependent upon the activity of the AtDGAT1.

The composition of the leaf oils in mock control leaves had 1.1% 18∶1 and no detectable levels of 20∶1 (Fig. 5C upper panel; and Table S1 for a complete range of fatty acids detected in the extracts). Co-infiltration of AtFAE1 and AtDGAT1 constructs without any VSP or hairpin resulted in 2.2% 18∶1 and 1.7% 20∶1, indicating that some overexpression of AtFAE1 is possible without any VSP added. The inclusion of hpNbFAD2 in combination with AtFAE1 and AtDGAT1 resulted in an increase of 18∶1 to 15.4% of the total fatty acid profile and 10.2% in 20∶1 in leaf oils. The inclusion of p19 in combination with hpNbFAD2, AtFAE1 and AtDGAT1 resulted in only 8% 18∶1, almost half of the oleic acid composition of the hairpin alone. Using V2 in combination with hpNbFAD2, AtFAE1 and AtDGAT1 resulted in 14.4% oleic acid and 13% 20∶1 in leaf oils. This level of 20∶1 is approximately 40% higher than that obtained using p19. It is surprising that hpNbFAD2 alone, with no VSP added, was also able to enhance shunting of lipids into new metabolites (Fig. 5C) although the total elongated products (20∶1+22∶1) is no higher than those resulting from the use of p19 (Table S1). The largest increase in leaf oil content (Fig. 5C lower panel) was with the combination of V2, hpNbFAD2, AtDGAT1 and AtFAE1, producing 14.1 µg/6 mg dry leaf tissue compared to assays with only the combination of AtFAE1 and AtDGAT1, producing 4.4 µg/6 mg dry leaf tissue (Fig. 3C). The combination of p19, hpNbFAD2, AtDGAT1 and AtFAE1 produced an oil content of 11.1 µg/6 mg dry leaf tissue and the combination of hpNbFAD2, AtDGAT1 and AtFAE1 generated an oil content of 9.5 µg/6 mg dry leaf tissue.

## Discussion

This study introduces new tools for the design and expression of complex metabolic engineering pathways in *N. benthamiana* that depend on both enhanced transgene overexpression and strong endogene silencing. A key tool introduced here is the VSP, V2, which outperformed p19 in our transient engineering of plant lipid metabolism. The lipid metabolite 18∶1 is a key intermediate substrate for a range of endogenous and transgenic reactions, and we used V2 and endogene silencing to shunt endogenous metabolism of 18∶1 into elongated fatty acids. V2 provided the best combination of endogene silencing and transgene overexpression, although p19 and a hairpin RNA alone were also able to support intermediate levels of metabolic flux for engineering of lipid pathways. To further aid metabolic engineering in *N. benthamiana*, we also provide a draft genome assembly currently spanning over 75% of the genome. Collectively the approaches outlined in this study can be used to support the elaborate engineering of metabolic pathways in transient leaf assays.

It is well documented that different plant viruses using their respective VSPs target different components of the host silencing apparatus [8]. V2 is one in this spectrum of VSPs. We have shown that V2 is able to support transgene overexpression yet also allow effective hpRNA-directed silencing of endogenes. This feature provides a distinct advantage over assays using p19, which significantly impaired the efficacy of the hpRNA (Fig. 5C). The design and molecular basis of silencing of endogenes triggered by hairpin silencing molecules is an important aspect of metabolic engineering [24]. Here we show that both 660 and 300 bp hairpin fragments are capable of effective silencing of the target gene, *NbFAD2.1*. The longer hairpin spanned regions of high homology with a second gene, *NbFAD2.2*, allowing cross-silencing, evidenced by both reductions in mRNA abundance and biochemical activities. These results indicate that single and multiple genes, that share sequence homology, can be accurately silenced depending of the design of the hairpin RNAi molecule.

The advent of low cost sequencing technologies allows large plant genomes, such as the 3.1 Gb genome of *N. benthamiana*, to be partially resolved in a relatively short period of time. Our assembly, covering more than 75% of the genome, represents a significant increase in the genomic resources for *N. benthamiana*. In this report, probing our DNA assembly with the FAD2 sequence from *A.thaliana* helped identify a two gene FAD2 family in *N. benthamiana*, namely *NbFAD2.1* and *NbFAD2.2*. These results were used to design a 660 bp hpRNA likely to cross-silence the FAD2 gene activities in leaves, and another shorter hairpin with more specific targeting of *NbFAD2.1*. Despite these advances, this draft genome assembly will continue to be improved as more sequence data becomes available. During the preparation of this report an independent sequencing effort announced their preliminary assembly of the *N. benthamiana* genome [25]. Ultimately, a range of data types will be needed to more fully resolve the genome sequence of this well-used plant.

Many fields of basic and applied plant biology require high-throughput screening methods to sift through candidate genes to find those most appropriate to the phenotype of interest. The benefit of low cost genomics and chemical gene synthesis now increases the need for expression formats that can readily assemble entire pathways into host cells with optimised substrate pools. V2-based assays now allow a rapid examination of transgenic pathways with optimised substrate pools and shunting of endogenous metabolites into engineered pathways that are dependent upon the silencing of endogenes. Therefore, V2-assays offer a dual capability for simultaneous transgene overexpression and endogene silencing, and significantly expands the number of applications to which transient assays can be applied. Although lipid metabolism is the focus of this study, we envision that V2-mediated assays will be useful for a range of basic and applied areas of plant research.

## Materials and Methods

### Plasmid Constructs for Transient Expression

Binary vectors were prepared by cloning the coding region of the gene into a modified version of the pORE04 binary vector described by Coutu et al. [26] in which the Cauliflower Mosaic Virus (CaMV) 35S promoter had been cloned into the SfoI site to yield 35S-pORE04. The coding sequences of p19 [2] and V2 [11] were chemically synthesised and cloned into 35S-pORE4 to yield pCW196 and pCW197, respectively. The AtFAE1 gene was chemically synthesised, however this ORF was impossible to clone directly into 35S-pORE4. We reasoned that this construct may be expressed at low levels in bacteria and be lethal. Therefore the catalase-1 intron was included in the 5′UTR during a subcloning step before ligation into a 35S expression vector to yield pCW483. The 35S binary expression construct for AtDGAT1 was used as described in Wood et al. [3]. The CaMV35S expression constructs of GFP and hpGFP, a hairpin against GFP, were described in Brosnan et al. [27]. The hpGFP construct was confirmed to include a 380 bp inverted repeat fragment targeting the first 380 bp of the GFP ORF. A 660 bp fragment of NbFAD2.1 was cloned by RT-PCR from leaf total RNA using primers designed against a DNA contig assembly containing NbFAD2 namely NbFAD2F1 5′ TAGAACAGATGGTGCACGACGT and NbFAD2R1 5′ TTATTGCGCACGAATGTGGCCA. The 660 bp NbFAD2 gene fragment was subsequently ligated into pENTR11 and recombined into the pHellsgate8 vector [28], using standard LR clonase reagents, to generate pFN033, a 35S-driven hairpin directed against NbFAD2.1 and NbFAD2.2, hpNbFAD2. A 300 bp fragment of NbFAD2.1 was also cloned in the same manner using 5′ CCTAAGCCGAAATCACAACTCG and 5′ TGGTACGCCATACATACACACGA and subsequently cloned into pHellsgate8 to generate pFN075, a 35S-driven hairpin directed against NbFAD2.1, hpNbFAD2-300.

### Isolation of Nuclear Genomic DNA Used for Deep Sequencing

The *N. benthamiana* used throughout this study is from an accession called ‘LabBenth’ that is routinely used at CSIRO, University of Sydney and New Zealand Institute of Plant and Food Research and available upon request. The nuclei were isolated as previously described [29], with the following modifications: 20 g of fully developed leaves were homogenized in a kitchen blender at maximum speed for 30 s in 300 mL ice cold nuclei isolation buffer (0.5 M mannitol, 10 mM PIPES, 10 mM MgCl_2_, 5 mM β-mercaptoethanol 2%, 10 mM sodium metabisulfite, polyvinylpyrrolidone (MW 40,000), 200 mM Lysine, 6 mM EGTA, pH 6). The homogenate was filtered through 4 layers of cheesecloth and 4 layers of miracloth. The filtrate was lysed by the gradual addition of Triton X-100 to a final concentration of 0.5% and the nuclei-enriched fraction collected by centrifugation (4°C; 15 min at 4967 *g*) using disposable 50 mL conical tubes. The pellets containing the nuclei were gently resuspended in the same volume of ice cold nuclei isolation buffer without β-mercaptoethanol, and centrifuged again. All pellets were resuspended and combined into 50 mL of the wash buffer and centrifuged again. The pellet was stored at −80°C prior to DNA extraction. The nuclear genomic DNA was extracted using a method originally optimised for RNA extraction from mango mesocarp [30] with some modifications as described in Hilario et al. [31]. The RNaseA treatment was performed during the lysis step and the phenol:chloroform extraction was omitted. The nuclear genomic DNA pellet was resuspended in 500 µL 10 mM Tris, 1 mM EDTA, pH 8 and centrifuged at 10000 *g* for 30 min to remove remnant starch granules. The starch-free supernatant containing nuclear genomic DNA was quantified by spectrophotometry at 260, 280 and 230 nm and stored at 4°C. A sample of DNA was analysed by pulse field gel electrophoresis to estimate the fragment size of the extracted sample at greater than ≥40 kbp.

Nuclear genomic DNA was sheared to an insert size of either ∼180 bp or ∼500 bp (Table 1) and prepared for paired-end sequencing on the Illumina HiSeq2000™ platform according to the manufacturer’s instructions. A LIMP (long insert matepaired end library) was also prepared according to manufacturer’s instructions and subsequently sequenced on one lane of the HiSeq2000™ platform. This library was subsequently analysed to contain ∼2000 bp inserts.

### Assembly of a Draft Genome

949,314,297 paired-end short-read sequences were obtained from the nine lanes (Table 1) of Illumina HiSeq2000™ data housed at the Australian Genome Research Facility (AGRF). Ten bases were removed from all reads following analysis using fastqc (http://www.bioinformatics.bbsrc.ac.uk/projects/fastqc). The read pairs (representing ∼48× coverage of the estimated genome size) were *de novo* assembled using the SOAPdenovo short-read assembler (version 1.05) [32] with a hash length of 65 and minimum scaffold length of 201 bases. The mate pair end data (∼2000 bp insert length) was trimmed to 36 bases in length, all duplicate reads removed and used to further scaffold the SOAPdenovo scaffolds using SSPACE (version 2.0 Basic; http://www.baseclear.com/) with the following parameter: insert size = 2000, range = 0.7, and orientation = RF. SOAPdenovo scaffolds were further subject to one iteration of gap closure using GapCloser (version 1.12; http://soap.genomics.org.cn).

### Cloning of the Full-length NbFAD2.1 and NbFAD2.2 Sequences from Genomic DNA

Based on deep sequencing data and related alignments the open reading frames of NbFAD2 sequences were PCR amplified from a genomic DNA template. *NbFAD2.1* was amplified using primers Forward 5′ TTTATGGGAGCTGGTGGTAATATGT and Reverse 5′ CCCTCAGAATTTGTTTTTGTACCAGAAA. *NbFAD2.2* was amplified using primers Forward 5′ TTTATGGGTGCTGGAGGTCGAA and Reverse 5′ CCCCTAATGAAGCTTGTTTTTATACC. The amplicon was subsequently sequenced using BigDye chemistry and verified to match those generated from deep sequencing efforts.

### Agrobacteria Infiltrations and N. benthamiana Growth Conditions

*Agrobacterium tumefaciens* strain AGL1 harbouring each binary vector was grown overnight at 28°C in LB broth supplemented with the appropriate antibiotics. Turbid cultures were supplemented with 100 µM acetosyringone and grown for a further 2 hours. Cultures were centrifuged (4000 *g* for 5 min at room temperature) and gently resuspended in infiltration buffer (5 mM MES, 5 mM MgSO_4_, pH 5.7, 100 µM acetosyringone) to an optical density ∼2.0. A final combination of cultures was prepared so that each *Agrobacterium* construct equalled OD 600 nm 0.3. The final mixture of *Agrobacterium* cells was infiltrated by the gentle squeezing of cultures from a 1 mL syringe barrel into the underside of fully-expanded leaves of 5 week-old *N. benthamiana* plants. Negative and mock control infiltation zones were used and refer to infiltrations using *Agrobacterium* cultures containing no binary vector. Plants were housed in a 24°C plant growth room with overhead lighting using 9∶15 light:dark cycle, where the light intensity was 400–500 µEinsteins m^−2^ s^−1^ at the leaf surface. Typically only two leaves per plant (each ∼12 cm in size) were used for infiltrations with non-ideal leaves (too old or too young) removed from the plant 1 day prior to the infiltration. Infiltrated areas of leaves, commonly 3 to 4 cm in diameter for oil quantification and ∼1.5 cm for total FAME profile analysis, were either circled by a permanent marker or identified by the GFP fluorescent signal using a hand-held NightSea (NightSea, Bedford, MA, USA) illumination system. A video outlining the infiltration procedure used in this study can be found at: http://www.youtube.com/watch?v=DhtZ0E6edcQ.

### GFP Fluorescence Imaging and Western Blot Analysis

GFP images were captured on a digital SLR (Nikon D60; 55–200 mm lens) using the NightSea fluorescent light and filter set (NightSea, Bedford, MA, USA). Infiltrated leaves that were still intact with the whole plant were photographed daily for 7 days. A 1 cm^2^ disc of infiltrated leaves were used for denaturing protein extraction and GFP was detected using an anti-GFP monoclonal antibody (1∶10000 dilution, Clontech) and goat anti-mouse HRP (1∶5000 dilution, Promega) using standard western blot techniques. Coomassie blue staining of total proteins in a duplicate gel was used as an indication of protein loading between samples.

### Lipid Analysis

Lipids were analysed as essentially as described previously [3]. All lipids were extracted from ∼10 mg dry weight (freeze dried overnight) of infiltrated leaf tissue using the method described by Bligh & Dyer [33]. For total lipid analysis an equivalent of 2 mg of dry weight leaf material was transmethylated using a solution of methanol/HCl/dichloromethane (10/1/1 by volume) at 80°C for 2 h to produce fatty acid methyl esters (FAME). The FAME were extracted in hexane, concentrated to near dryness under a stream of N_2_ gas and quickly reconstituted in hexane prior to analysis by GC. TAG fractions were separated using a 1-phase TLC system on pre-coated silica gel plates (Silica gel 60, Merck). A sample equivalent to 6 mg dry weight of leaf tissue was run using hexane/diethyl ether/acetic acid (70/30/1 by volume). The lipid spots and appropriate standards, were visualised by brief exposures to iodine vapour, collected into vials and transmethylated to produce FAME for GC analysis as described above. Analysis of the leaf phosphatidylcholine (PC) fraction was performed as previously described [3] using a two-dimensional TLC system.

GC was performed using an Agilent Technologies 6890N GC (Palo Alto, California, USA) equipped with a non-polar Equity™-1 fused silica capillary column (15 m x 0.1 mm i.d., 0.1 µm film thickness), an FID, a split/splitless injector and an Agilent Technologies 7683 Series autosampler and injector using helium as the carrier gas. Samples were injected in splitless mode at an oven temperature of 120°C and after injection the oven temperature was raised to 201°C at 10°C.min^−1^ and finally to 270°C and held for 20 min. Peaks were quantified with Agilent Technologies ChemStation software (Rev B.03.01 (317), Palo Alto, California, USA). Peak responses were similar for the fatty acids of authentic Nu-Check GLC standard-411 (Nu-Check Prep Inc, MN, USA) which contains equal proportions of 31 different fatty acid methyl esters, including 18∶1, 18∶2 and 20∶1. Slight variations of peak responses among peaks were balanced by multiplying the peak areas by normalization factors of each peak. The proportion of each fatty acid in total fatty acids was calculated on the basis of individual and total peaks areas of the fatty acids.

### Quantitative Real-Time Analysis

Total RNA extracted from infiltrated leaf tissue using Qiagen RNeasy Plant Mini Kit according to the manufacturer’s protocol including DNase treatment. 360 ng of extracted total RNA was reverse transcribed using SuperScript™ III First-Strand Synthesis System for RT-PCR (Invitrogen). The primers used for amplification of 205 bp fragment of NbFAD2.1, Forward 5′ CACACTACAATGCAATGGAGG and Reverse 5′ CCAAAGACCAATACCAAATTCC. The primers used for amplification of 148 bp fragment of NbFAD2.2 Forward 5′ AGAGAAGCAAGGGAATGTGTTTAC and Reverse 5′ AGCAAAGCCTAAAACTTCCCAG. Endogenous glyceraldehyde-3-phosphate dehydrogenase (*NbGAPDH*) was selected as the reference gene. qRT-PCR was performed in a 96 well format in the BIO-RAD CFX96 Real-Time System (BioRad Laboratories). The thermal profile of the qRT-PCR procedure was set to 95°C for 3 min followed by 39 repeated cycles of: 10 s at 95°C, 30 s at 60°C and 30 s at 68°C. Melt curve analysis performed from incubation at 65°C to 0.5°C incremental ramp up to 95°C. Melting curves were used to validate product specificity. All samples were amplified in triplicates from the same total RNA preparation and the mean value was used for further analysis. Primer efficiencies were also determined for each gene and each primer pair was 99.9–100% efficient.

### Experimental Design and Statistical Analysis of Leaf assays

Leaf assays for metabolic engineering of lipid profiles were conducted on a minimum of four and maximum of ten leaves with a minimum of four and maximum of ten independent infiltration zones per leaf. Preliminary experiments determined that all regions of leaves performed at similar levels in metabolic engineering scenarios, except for a ∼1 cm wide band across the very tip of each leaf and this region was avoided in subsequent infiltrations (data not shown). The location of spots on leaves and plants were recorded and data were analysed with a linear mixed model in GenStat™ (www.GenStat.co.uk), accounting for possible random plant-to-plant and leaf-to-leaf effects. All data were transformed (square root) prior to analysis to achieve constant variance in the residuals, so that all quoted means, standard errors and least significant differences (LSDs) are those calculated from the transformed data. In the figures, the means and (5%) LSD bar limits have been back-transformed to the scale of the original data. The LSD bars in the figures provide a means to determine statistical significance visually - when the LSD bars for two metabolites do not overlap, the metabolites are significantly different at the *p* = 0.05 level.

## Supporting Information

Figure S1 **Relative expression of** ***NbFAD2.1*** **compared to** ***NbFAD2.2*** **in** ***N. benthamiana*** **leaves.** Expression levels of *NbFAD2.1* and *NbFAD2.2* measured in mid-size *N. benthamiana* leaves. Total RNA extracted from at least 3 leaves.
(TIF)

Figure S2 **Alignment of hpNbFAD2 and hpNbFAD2-300 on** ***NbFAD2.2.*** High homologous regions between hpNbFAD2 and *NbFAD2.2* support cross silencing of *NbFAD2.1* and *NbFAD2.2* from one hairpin construct. There are relatively less homologous regions between the shorter hairpin hpNbFAD2-300 and NbFAD2.2.
(TIF)

Table S1 **The complete fatty acid profile of triacylglyceride (TAG) fraction from infiltrated leaves.** Leaves infiltrated with combinations of hpNbFAD2, V2 or p19, and a two-step metabolic pathway, *AtFAE1* and *AtDGAT1*, for production of modified oils. The table outlines the changes in endogenous metabolite levels and the production of elongated products due to expression of *AtFAE1*. Error bars represent the standard error of the mean, *p* = 0.05%.
(TIF)
