# Supplementary figures and images for: Advanced Engineering of Lipid Metabolism in Nicotiana benthamiana Using a Draft Genome and the V2 Viral Silencing-Suppressor Protein
Source: PLoS One. 2012 Dec 26;7(12):e52717. doi: 10.1371/journal.pone.0052717 (PMC3530501; doi:10.1371/journal.pone.0052717)

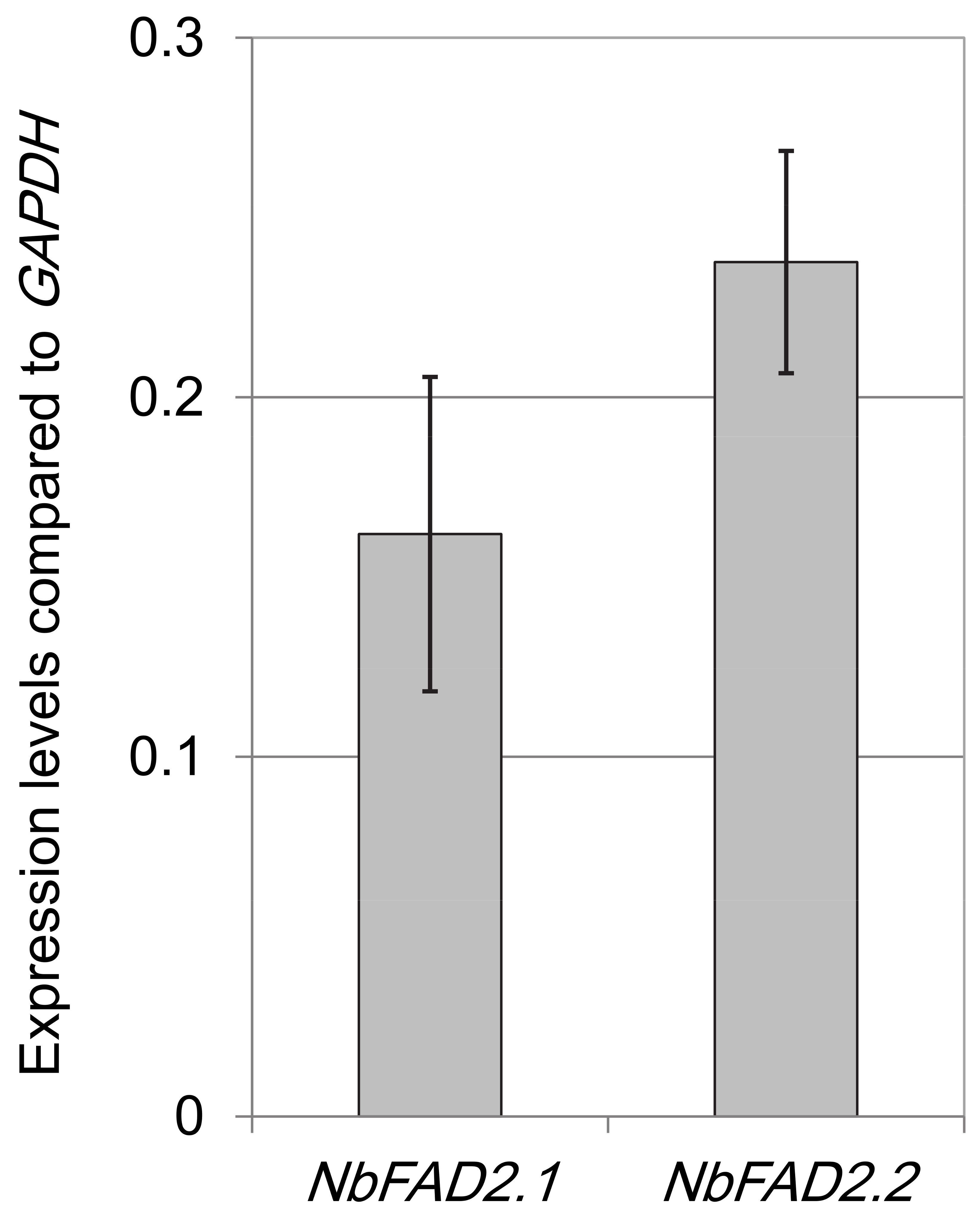

Supplement: Figure S1 — Relative expression of NbFAD2.1 compared to NbFAD2.2 in N. benthamiana leaves. Expression levels of NbFAD2.1 and NbFAD2.2 measured in mid-size N. benthamiana leaves. Total RNA extracted from at least 3 leaves. (TIF) [file pone.0052717.s001.tif]

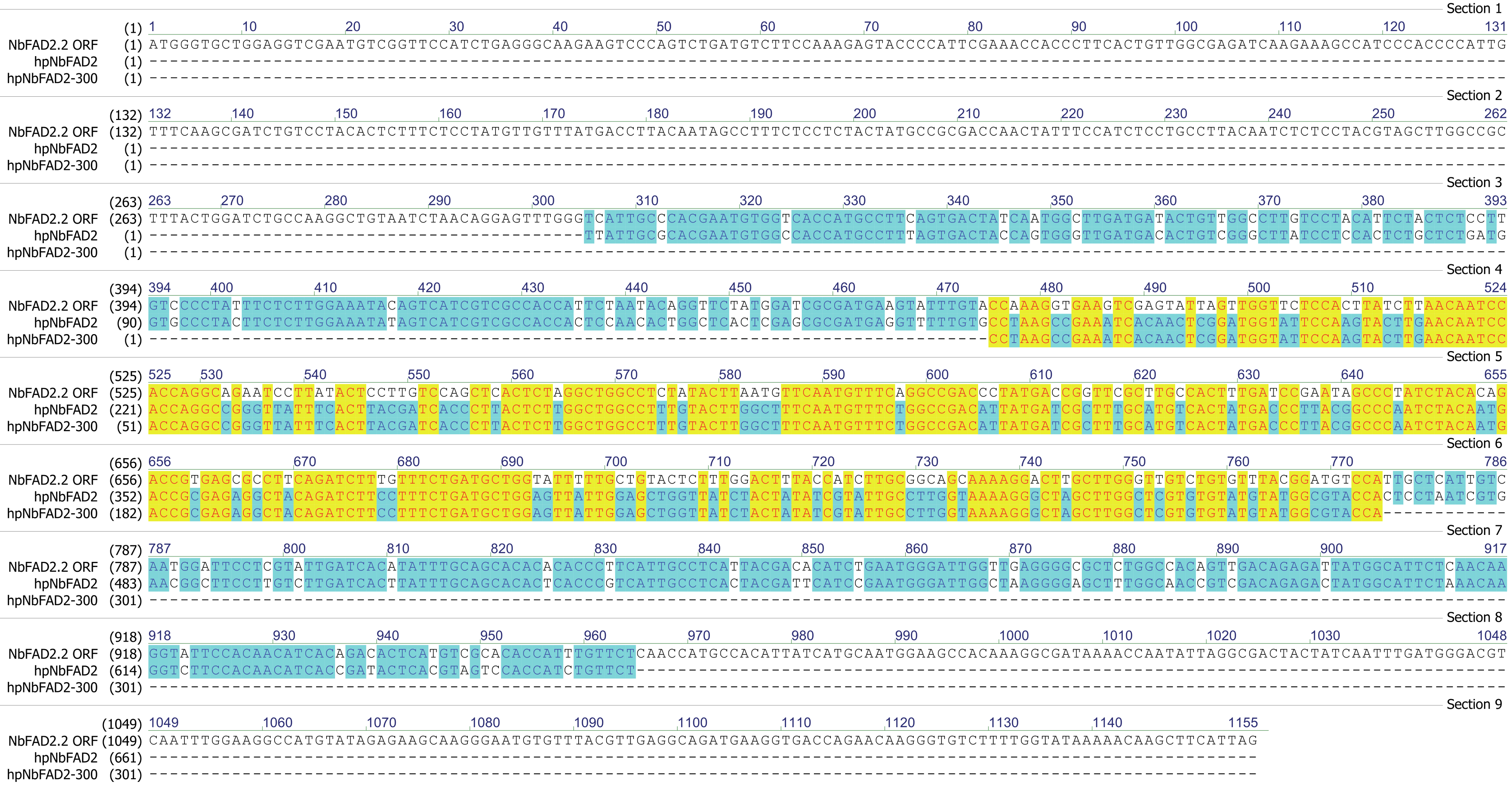

Supplement: Figure S2 — Alignment of hpNbFAD2 and hpNbFAD2-300 on NbFAD2.2. High homologous regions between hpNbFAD2 and NbFAD2.2 support cross silencing of NbFAD2.1 and NbFAD2.2 from one hairpin construct. There are relatively less homologous regions between the shorter hairpin hpNbFAD2-300 and NbFAD2.2. (TIF) [file pone.0052717.s002.tif]

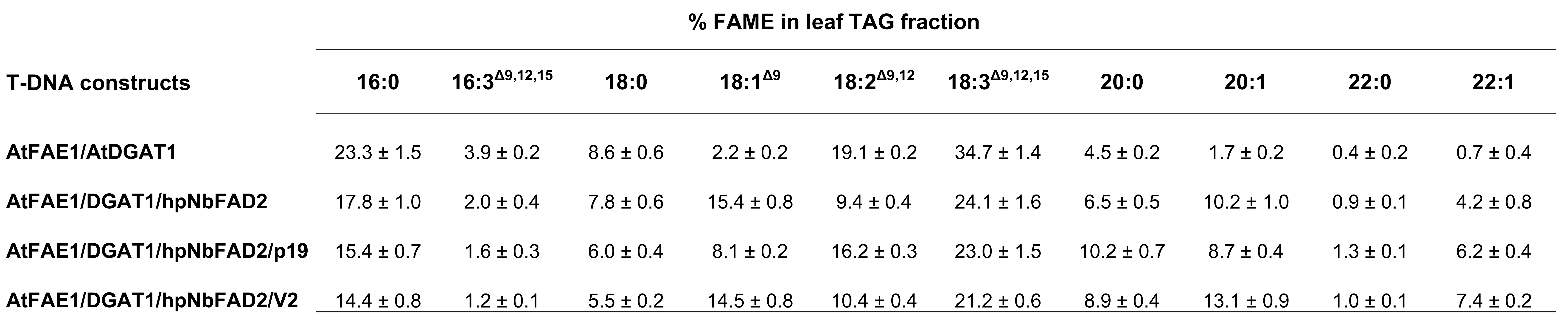

Supplement: Table S1 — The complete fatty acid profile of triacylglyceride (TAG) fraction from infiltrated leaves. Leaves infiltrated with combinations of hpNbFAD2, V2 or p19, and a two-step metabolic pathway, AtFAE1 and AtDGAT1, for production of modified oils. The table outlines the changes in endogenous metabolite levels and the production of elongated products due to expression of AtFAE1. Error bars represent the standard error of the mean, p = 0.05%. (TIF) [file pone.0052717.s003.tif]
